# Large-scale reference-free analysis of flavivirus sequences in *Aedes aegypti* whole genome DNA sequencing data

**DOI:** 10.1186/s13071-023-05898-8

**Published:** 2023-08-05

**Authors:** Anton Spadar, Jody E. Phelan, Taane G. Clark, Susana Campino

**Affiliations:** 1 Faculty of Infectious and Tropical Diseases, London School of Hygiene and Tropical Medicine, London, UK; 2 Faculty of Epidemiology and Population Health, London School of Hygiene and Tropical Medicine, London, UK; 3 Department of Infection Biology, London School of Hygiene and Tropical Medicine, Keppel Street, London, WC1E 7HT UK

**Keywords:** Mosquito, Aedes, Flavivirus, Arbovirus, Endogenous viral element, nrEVE

## Abstract

Flaviviruses are a diverse group of RNA viruses, which include the etiological agents of Zika, dengue and yellow fever that are transmitted by mosquitoes. Flaviviruses do not encode reverse transcriptase and cannot reverse transcribe into DNA, yet DNA sequences of flaviviruses are found both integrated in the chromosomes of *Aedes aegypti* mosquitoes and as extrachromosomal sequences. We have previously examined the *Ae. aegypti* reference genome to identify flavivirus integrations and analyzed conservation of these sequences among whole-genome data of 464 *Ae. aegypti* collected across 10 countries globally. Here, we extended this analysis by identifying flavivirus sequences in these samples independently of the *Ae. aegypti* reference assembly. Our aim was to identify the complete set of viral sequences, including those absent in the reference genome, and their geographical distribution. We compared the identified sequences using BLASTn and applied machine learning methods to identify clusters of similar sequences. Apart from clusters of sequences that correspond to the four viral integration events that we had previously described, we identified 19 smaller clusters. The only cluster with a strong geographic association consisted of Cell-fusing agent virus-like sequences specific to Thailand. The remaining clusters did not have a geographic association and mostly consisted of near identical short sequences without strong similarity to any known flaviviral genomes. The short read sequencing data did not permit us to determine whether identified sequences were extrachromosomal or integrated into *Ae. aegypti* chromosomes. Our results suggest that Liverpool strain and field *Ae. aegypti* mosquitoes have a similar variety of conserved flaviviral DNA, whose functional role should be investigated in follow-up studies.

**Graphical Abstract:**

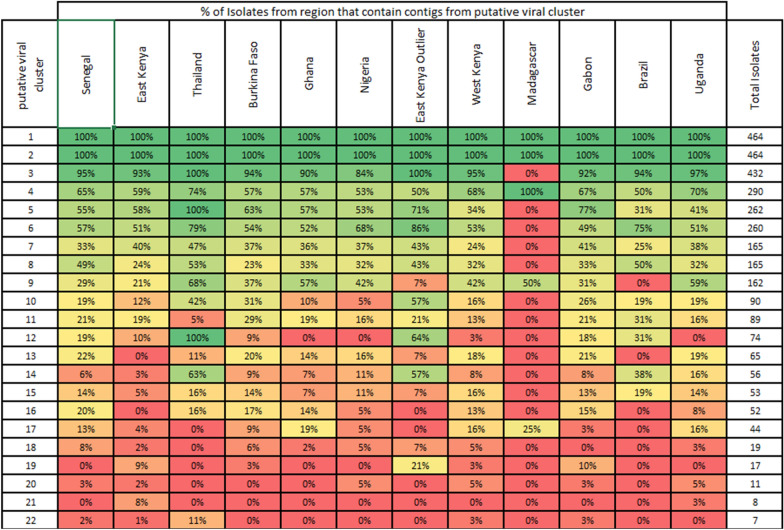

**Supplementary Information:**

The online version contains supplementary material available at 10.1186/s13071-023-05898-8.

In our previous publication [1], we have examined reference genomes of *Aedes aegypti* and *Ae. albopictus* to identify integrated flavivirus sequences to improve our understanding of non-retroviral endogenous viral elements (nrEVEs) (see [2] for recent review). We have also examined the publicly available *Aedes* whole-genome sequencing data to understand conservation of the identified viral sequences. We have found that nrEVEs in *Ae. albopictus* were very diverse with little conservation. In contrast, we found that nearly all nrEVEs in the *Ae. aegypti* reference genome originated from four distinct viral integration events (VIEs). We concluded that the diversity of flaviviral nrEVE sequences in the reference genome is the result of duplication and fragmentation of these four VIEs. The *Ae. aegypti* nrEVE fragments were present in almost all examined sequenced isolates but showed a star-like phylogenetic structure without clades, indicative of a recent population expansion event from a common ancestor.

Alongside these four core events, in our previous work we observed many short (< 50 nt) flavivirus-like sequences in the *Ae. aegypti* reference genome, which we did not analyse at the time [1]. In addition, other investigations have found long sequences with very high identity (> 95%) to a Cell-fusing agent virus (NC_001564.2), with some geographic specificity [3, 4]. Other studies have found that viral DNA is generated by *Aedes* mosquitoes during flavivirus infection [5, 6].

The limitation of our previous approach was the focus on nrEVEs present in mosquito reference genomes. In this brief report, we address this limitation by examining the landscape of putative flaviviral DNA (pfDNA) in *Ae. aegypti* independently of reference genome. Specifically, we aimed to identify any geographically specific pfDNA that is absent in the reference genome. In a robust analysis, we also deliberately included high-quality sequences with very short match length between mosquito DNA and viral sequences to strengthen any conserved sequence signals across geographic regions. As we are using short-read sequencing data, we are not able to determine whether pfDNA sequences are intra- or extrachromosomal. We also do not re-examine the sequences that belong to the four viral integration events (VIE1 to VIE4), as described previously [1]. For our analysis we aligned 464 publicly available *Ae. aegypti* whole-genome sequencing (WGS) libraries [7] to all NCBI RefSeq database Flaviviridae sequences (*n* = 118, as of April 2020) [7] and nrEVEs we previously identified [1] (Table 1, Additional file 2: Fig. S1). For the alignment, we used bowtie2 software [8] with very sensitive settings (-D 15 -R 2 -N 0 -L 11 -i S, 1, 0.75). The mapped reads were assembled de novo using SPAdes (v3.13.0) software [9] into 25,049 contigs, with a median number of 53 contigs per isolate (range: 8–138). The distribution of contig lengths followed an exponential-like distribution with median length of 200 nt and a longest contig of 10,057 nt.

**Table 1:**
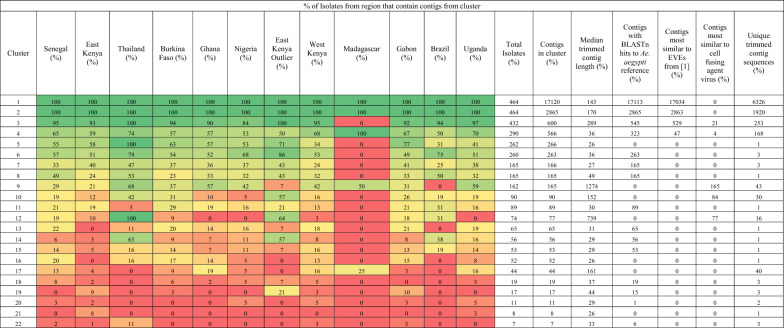
Characteristics of all clusters

The disaggregated data and nucleotide sequences are in Additional file 3: Table S1

Due to the size of the dataset, the contigs were analyzed programmatically instead of following the more detailed manual approach we used previously [1]. We focused on the sequence similarity (measured by BLASTn *e*-value) between pairs of contigs to understand how the pfDNA sequences within and between samples relate to each other. The raw contigs consisted of both viral and non-viral sequences, but we trimmed the latter to avoid clusters being inferred because of the non-viral sequences. We used BLASTn v2.9.0 [10] (word-size 11, *e*-value cutoff 0.0001 throughout) to identify parts of contigs with similarity to Flaviviridae reference genomes. We also used BLASTn to identify parts of contigs with similarity to the reference genome of *Danio rerio* (GCF_000002035.6), which was our proxy for generic sequences (e.g. homopolymers or short repeats) as this is a high-quality reference and belongs to a different phylum. We trimmed the initial contig by removing the sequences that either had no similarity to viral sequences or after those that had a match against *D. rerio*, our proxy for generic sequences.

After trimming, 22,764 contigs with at least 25nt in length were carried forward for cluster analysis. We created a similarity matrix for the trimmed contigs based on pairwise BLASTn e-values. The matrix value *(i,j)* is the lowest *e*-value from the comparison of contigs *i* and *j*. For the contig pairs without matches, e-values were set to 1. We used the UMAP software [11], a dimensional reduction technique, to represent the similarity matrix in two dimensions (Fig. 1). Clusters of contigs in the 2D representation were subsequently detected using HDBSCAN software [12]. All contigs were assigned to one of the clusters, and these clusters were the focus of the subsequent analysis (Table 1, Additional file 3: Table S1). Because we grouped sequences based on pairwise e-values, the grouping is independent of similarity of sequences to publicly available Flaviviridae reference genomes. As described later, in nearly all cases the similarity of the contig to known flaviviruses was too low to suggest which virus was the source of pfDNA.

**Fig. 1:**
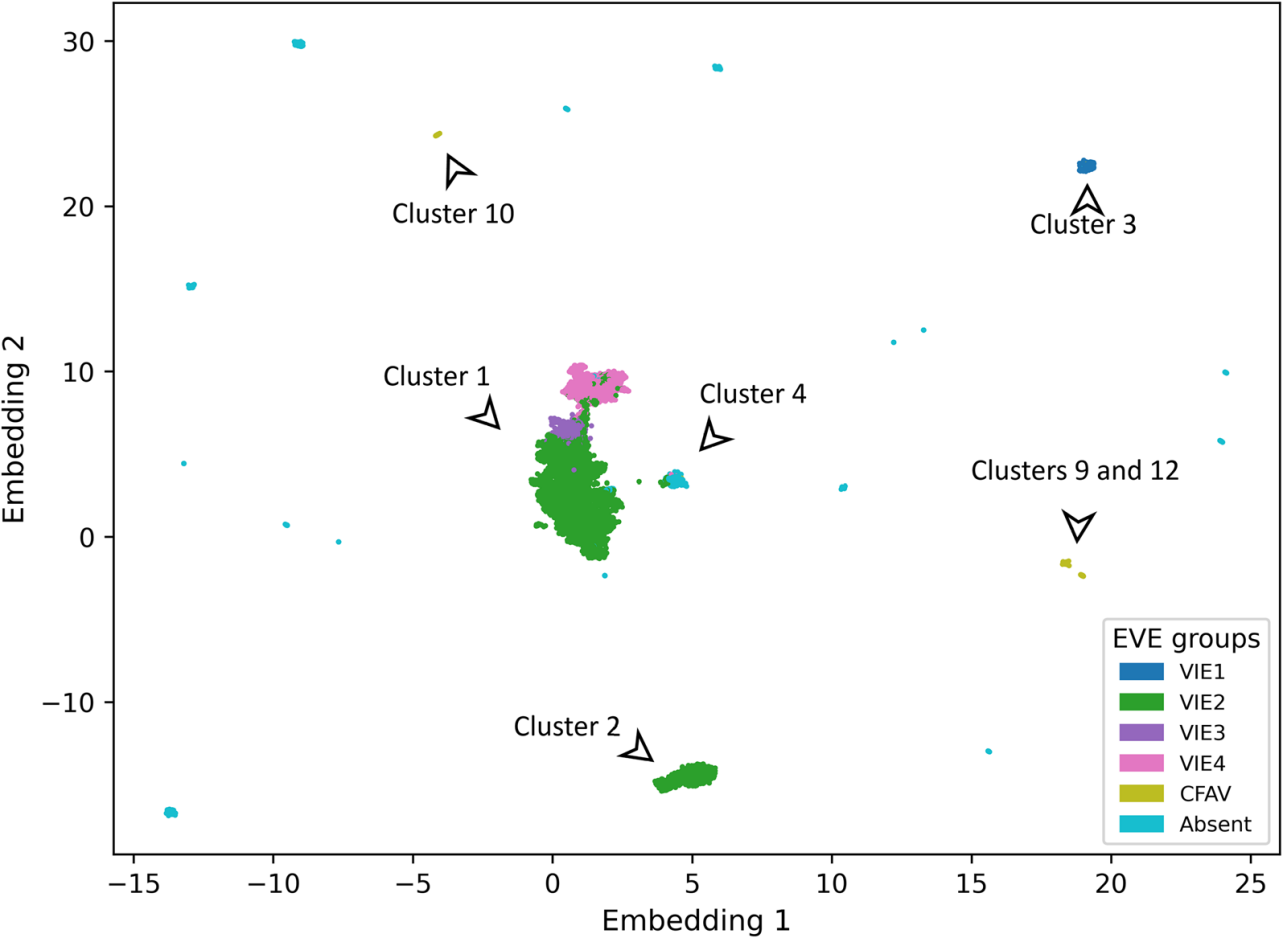
Dimensional reduction of all-vs.-all comparison of contigs. Each point is a contig with color based on the nrEVE or CFAV with the lowest BLASTn *e*-value. Axes are dimensionless, and only key clusters are labeled

Most pfDNA clusters identified (Table 1) could be separated into three main categories: universal nrEVEs identified previously from the reference assembly, Cell-fusing agent-like sequences described previously, and groups of short (< 50 nt) near-identical sequences [1, 3, 4]. The first category (universal nrEVEs) consists of clusters 1 and 2 (Fig. 1), and every sample in our study had contigs in each of these clusters. Cluster 1 is the largest and consists of 75.2% (*n* = 17,120) of all contigs. It represents what we have previously termed viral integration events VIE2, VIE3, and VIE4 [1]. Cluster 2 is second largest and consists of 12.6% (*n* = 2865) of all contigs. Cluster 2 represents reference assembly sequences we previously termed AE16.2 and AE17.2, which are a subset of VIE2. The distinct nature of VIE2, VIE3 and VIE4 that we have previously described was visible from their distinct localization in cluster 1 (Fig. 1). In brief, we have previously found that these VIEs originated from three distinct viral integration events. The original integrated sequences have subsequently undergone duplication and fragmentation leading to observed diversity of sequences. A fuller discussion of these clusters can be found in our previous work [1].

Cluster 3 is the next largest. It consists of 2.6% (*n* = 600) of all contigs, and nearly all (*n* = 432/464) isolates had a contig belonging to this cluster. Madagascar was the only country completely missing from this cluster. The trimmed contigs were between 32 and 328 nt long with a median length of 289 nt. The longest of these contigs shares ~ 71% identity and 95% coverage with a 9738–10,050-nt region of the Calbertado virus genome (KX669684.1). VIE1, which we previously identified, also maps to these sequences but with much higher identity (> 97%) compared to Calbertado virus [1]. Thus, cluster 3 corresponds to what we previously termed VIE1. Based on our analysis here, the *Ae. aegypti* AaegL5 reference assembly has only half of the VIE1 sequence. This result is consistent with our previous observation that VIE1 is less conserved than VIE2, VIE3 or VIE4 [1].

Another category of clusters consisted of clusters 9, 10 and 12 with 0.7% (*n* = 165), 0.4% (*n* = 90) and 0.3% (*n* = 77) of contigs which were previously described (Table 1) [3, 4]. Our BLASTn search of the trimmed contigs from these clusters against AaegL5 reference genome returned no hits; however, the sequences showed strong similarity to a Cell-fusing agent virus (NC_001564.2) with average identity of 94.4% and 96.2% for clusters 9 and 12, and 74.4% for cluster 10 (Fig. 2, Additional file 3: Table S1). Clusters 9 and 12 are the only ones where the closest known matching virus, a Cell-fusing agent virus, may be the source of pfDNA. The sequence diversity in these clusters was limited with 43, 30 and 16 unique sequences in clusters 9, 10 and 12, respectively. Notably, cluster 12 has limited geographic distribution but includes all Thai samples (*n* = 19) as well as 9 samples from East Kenya. The latter has a known phylogenetic link to the Thai *Ae. aegypti* population [13].

**Fig. 2:**
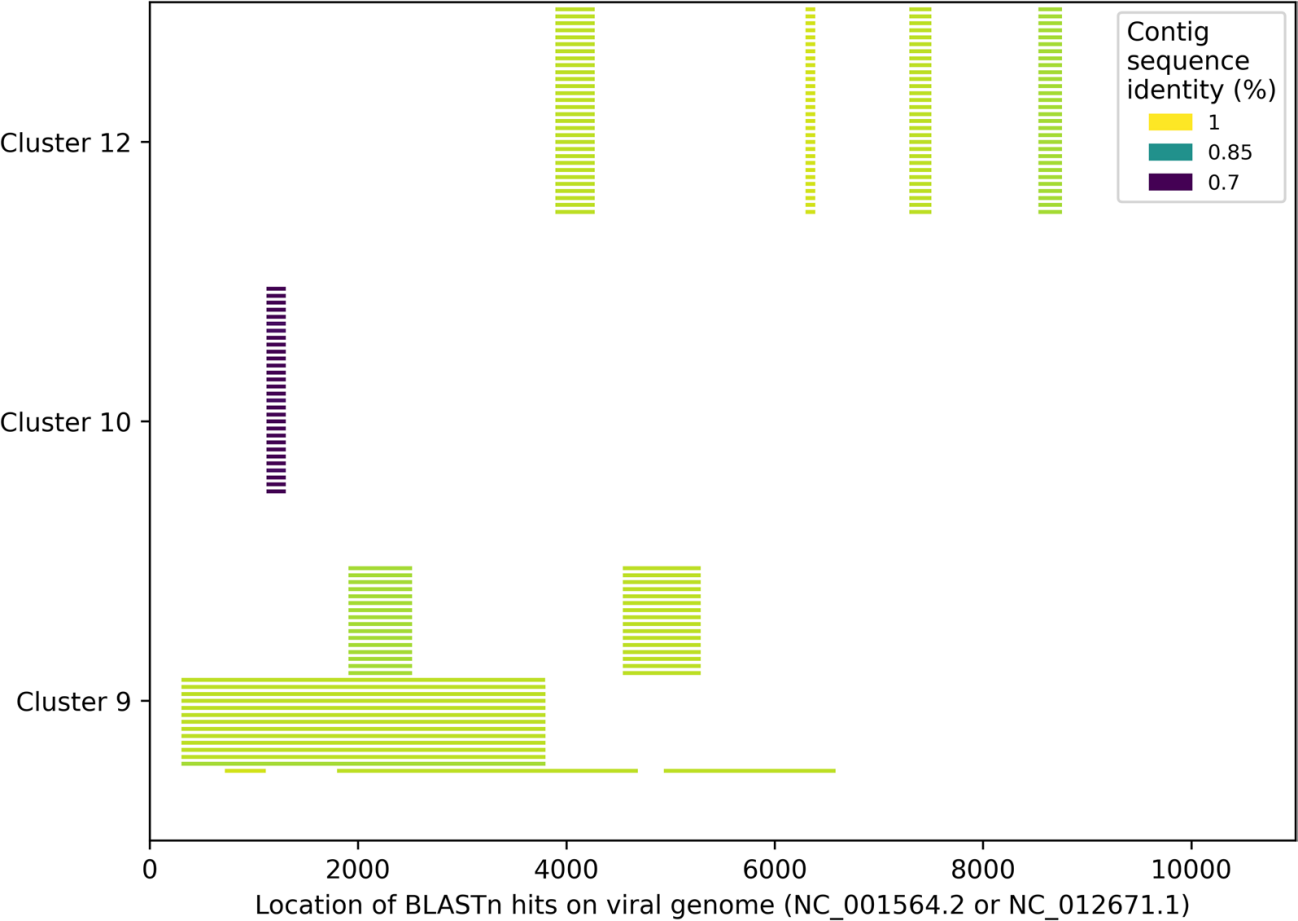
Location of the BLASTn hits against the best matching viral genome. Contigs from clusters 9 and 12 (map to a Cell-fusing agent virus) and cluster 10 (mapping Quang Binh virus). Only 30 contigs per cluster are shown selected based on the longest total length of BLASTn hits against viral genome

Cluster 17 contained 40 unique viral sequences from 44 isolates. All sequences map to a region between 3 and 255 nt of Falli virus (MN567479.1) with 70% identity. While the lengths of viral sequences vary between 94 and 261 nt, all shorter ones are sub-sequences of the longest sequence with > 95% identity. The cluster was most prevalent in Ghana (19%) and Senegal (13%).

Clusters 5–8, 11, 13–16, and 18–22 all consist of short (< 50 nt) sequences, each with 1–3 unique sequences (Table 1). There was nothing notable about these clusters, and we believe they are spurious hits. In contrast to these clusters, all other clusters [1–4, 9, 10, 12, 17] had at least 35 sequences > 100 nt (Additional file 3: Table S1). This bifurcation of clusters into those that contain only sequences < 50 nt and those that include sequences > 100 nt serves to separate white noise from genuine hits.

Finally, cluster 4 [3, 4] is an analytical artifact. It contains 566 contigs with 168 unique pfDNA sequences. A minority of these contigs (*n* = 46/566) belonged to three Gabonese samples (SRR11006792, SRR11006794, SRR11006795) and are part of previously described VIE4 and VIE2 based on > 95% identity to sequences of these VIEs. Consequently, these 46 contigs also had hits against the *Ae. aegypti* reference genome. In addition, all four Madagascan samples had an identical 606-nt sequence that matched a 743–1349-nt region of a Cell-fusing agent virus with 86% identity. The remaining 516 contigs in the cluster had short (25–72 nt) hits against the viral genome and similar length hits (28–72 nt) against the *Ae. aegypti* reference genome. In post-clustering quality control, we found that clustering of these sequences was a statistical artifact. The e-value for pairs of contigs without any BLASTn hit was set to 1.0, and as a result the main similarity between these 516 contigs was dissimilarity to other contigs.

Despite extensive analysis, it is possible that further pfDNA are present in the 464 isolates we examined. Our analysis did not find extensive diversity or geographic patterns one might expect if pfDNA played an adaptive immunity-like function in *Ae. aegypti* [2, 14–16]. However, this does not rule out that pfDNA may have an immunity-like function. In addition, previous research [3, 4] detected limited geographic pfDNA specificity that we also reconfirmed in our results. None of the 14 small clusters composed of short near-identical sequences appears to have diversity resembling the diversity of flaviviruses that are known to infect *Ae. aegypti*. The major clusters 1, 2, and 3 were universally present. Notably, their phylogenetic trees have a star-like appearance we identified previously [1], which can be caused by weak phylogenetic signals. As has been demonstrated, pfDNAs may impact viral titers [3], as does manipulation of si- and pi-RNA pathways [17–20]. There is some published evidence of interference with si- or pi-RNA pathways resulting in clear increases in mortality or other serious negative fitness effects in mosquito [6, 21]. However, other investigations [22–25] have reported decreased mosquito fitness and/or fecundity following inhibition of flavivirus infection. While the evidence is tenuous, at least in *Ae. aegypti,* insect-specific flaviviruses may be symbiotic. If this hypothesis is correct, it should be considered when developing strategies to control arboviral infections via genetic engineering on mosquitoes [e.g. engineered microRNAs (miRNAs) targeting specific flaviviruses]. Further work with *Ae. albopictus* mosquitoes, not examined here may provide different results due to much higher diversity of pfDNA in that species [1]. Moving forward, large-scale sequencing of both *Ae. albopictus* and *Ae. aegypti* across unrepresented populations is required to provide a more comprehensive global picture of pfDNA distribution, ultimately leading to further insights into important vector and viral biology.

### Supplementary Information

**Additional file 1.** Analysis input data and scripts.

**Additional file 2.** Analysis workflow.

**Additional file 3.** Identified contigs of possible viral origin and cluster assignment.

## Data Availability

All sequence data are available from NCBI. Scripts and data are available at in Additional file 1: File S1.
