# Supplementary figures and images for: Large-scale reference-free analysis of flavivirus sequences in Aedes aegypti whole genome DNA sequencing data
Source: Parasit Vectors. 2023 Aug 5;16:265. doi: 10.1186/s13071-023-05898-8 (PMC10403824; doi:10.1186/s13071-023-05898-8)

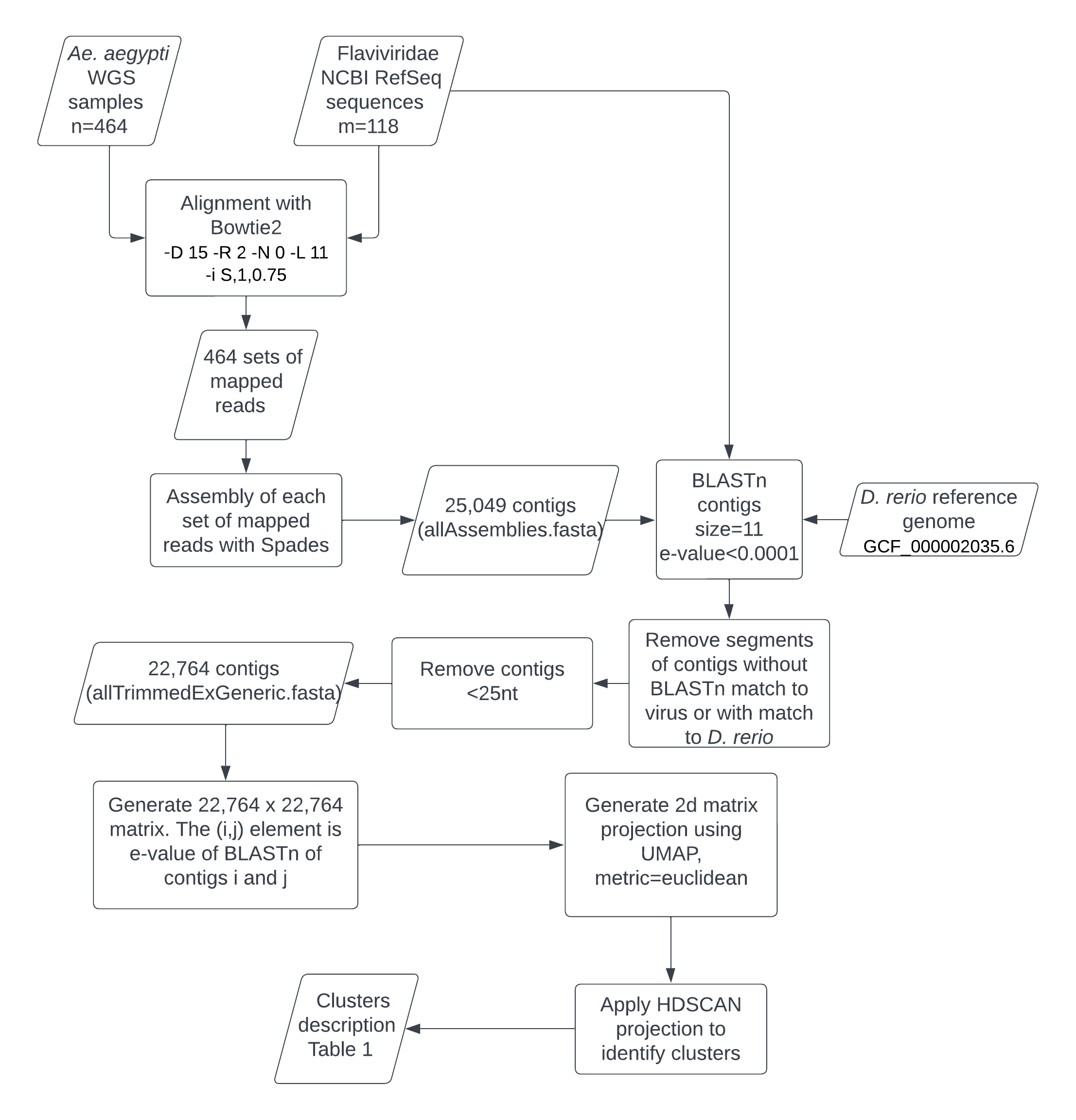

Supplement: Supplementary file 2 — Additional file 2. Analysis workflow. [file 13071_2023_5898_MOESM2_ESM.png]
